# Effect of running therapy on depression (EFFORT-D). Design of a randomised controlled trial in adult patients [ISRCTN 1894]

**DOI:** 10.1186/1471-2458-12-50

**Published:** 2012-01-19

**Authors:** Frank R Kruisdijk, Ingrid JM Hendriksen, Erwin CPM Tak, Aartjan TF Beekman, Marijke Hopman-Rock

**Affiliations:** 1 GGZ Centraal Centers for Mental Health, Symfora-Meander Centre for Psychiatry, Utrechtseweg 266, 3800 DB Amersfoort, The Netherlands; 2 TNO Expert Center Life Style, Wassenaarseweg 56, 2333 AL Leiden, The Netherlands; 3 Body@Work, Research Center Physical Activity, Work and Health, TNO-VUmc, Van der Boechhorststraat 7, 1081 BT Amsterdam, The Netherlands; 4 Department of Psychiatry, VU University Medical Centre, A.J. Ernststraat 887, 1081 HL Amsterdam, The Netherlands; 5 The EMGO Institute for Health and Care Research (EMGO+), VU University Medical Centre, Van der Boechhorststraat 7, 1081 BT Amsterdam, The Netherlands

## Abstract

**Background:**

The societal and personal burden of depressive illness is considerable. Despite the developments in treatment strategies, the effectiveness of both medication and psychotherapy is not ideal. Physical activity, including exercise, is a relatively cheap and non-harmful lifestyle intervention which lacks the side-effects of medication and does not require the introspective ability necessary for most psychotherapies. Several cohort studies and randomised controlled trials (RCTs) have been performed to establish the effect of physical activity on prevention and remission of depressive illness. However, recent meta-analysis's of all RCTs in this area showed conflicting results. The objective of the present article is to describe the design of a RCT examining the effect of exercise on depressive patients.

**Methods/Design:**

The EFFect Of Running Therapy on Depression in adults (EFFORT-D) is a RCT, studying the effectiveness of exercise therapy (running therapy (RT) or Nordic walking (NW)) on depression in adults, in addition to usual care. The study population consists of patients with depressive disorder, Hamilton Rating Scale for Depression (HRSD) ≥ 14, recruited from specialised mental health care. The experimental group receives the exercise intervention besides treatment as usual, the control group receives treatment as usual. The intervention program is a group-based, 1 h session, two times a week for 6 months and of increasing intensity. The control group only performs low intensive non-aerobic exercises. Measurements are performed at inclusion and at 3,6 and 12 months.

Primary outcome measure is reduction in depressive symptoms measured by the HRSD. Cardio-respiratory fitness is measured using a sub maximal cycling test, biometric information is gathered and blood samples are collected for metabolic parameters. Also, co-morbidity with pain, anxiety and personality traits is studied, as well as quality of life and cost-effectiveness.

**Discussion:**

Exercise in depression can be used as a standalone or as an add-on intervention. In specialised mental health care, chronic forms of depression, co-morbid anxiety or physical complaints and treatment resistance are common. An add-on strategy therefore seems the best choice. This is the first high quality large trial into the effectiveness of exercise as an add-on treatment for depression in adult patients in specialised mental health care.

**Trial registration:**

Netherlands Trial Register (NTR): NTR1894

## Background

Depression is a common disorder. The lifetime prevalence of depressive disorders in Dutch adults is 19% [1]. Recurrence of symptoms occurs in an unfavourable and fluctuating course in 44% and a severe chronic course in 32% of the patients. Depressive disorders obviously have negative effects on wellbeing and daily personal and professional functioning.

### Antidepressants and psychotherapy

Treatment frequently includes prescription of antidepressants. However, the effectiveness of such treatment may be limited because of poor compliance and limited effectiveness. A recent analysis of data of the US Food and Drugs Administration, showed relatively small drug-placebo differences in antidepressant efficacy [2]. Other disadvantages of antidepressants are unpleasant side effects and increased risk of hypertension in depressed patients combined with Diabetes Mellitus type II (DM II) [3,4].

A systematic review of randomised controlled trials into effectiveness and cost-effectiveness of brief psychological treatment for depression showed that some forms, especially cognitive-behavioural based approaches, were beneficial in the treatment of outpatients [5]. A meta-analysis, regarding a mostly adult population, found a favourable outcome for a combination of psychotherapy and pharmacotherapy compared to psychological treatment alone for depression in the short-term [6]. A recent meta-analysis examined whether the quality of the studies investigating psychotherapy for adult depression was associated with the effect-size found in these studies [7]. It showed that the effects of psychotherapy for adult depression have been overestimated in meta-analytical studies. The authors stated that the effects of psychotherapy are much smaller than is assumed.

The traditional treatment strategies, medication and psychotherapy, are still not ideal and a new intervention, exercise, focusing on a different psychopathological mechanism of depression is needed.

### Exercise

Exercise is a potential alternative low-cost therapy, but more studies are needed to define the place of exercise in a stepped care program for depression [8]. Exercise is relatively safe and has less negative side effects than antidepressants. Obviously, exercise has also many beneficial effects on physical health [9,10], and is expected to have additional advantages in depressed patients who suffer from a combination of mental and physical problems, such as pain complaints or increased risk of cardiac morbidity. Exercise is suitable for most individuals, participating in an exercise program promotes social integration and successful adaptation can increase self-esteem. Finally, exercise may reduce the negative side effects of antidepressants, such as fatigue, thus increasing compliance in medication use.

### Scientific evidence for the efficacy of exercise

Recent reviews and meta-analyses suggest that exercise leads to improvements in depressive symptoms [11,12]. Following the cumulative evidence from prospective cohort studies and RCTs, exercise has proven *protective *benefits for several aspects of mental health in general and for symptoms of depression in particular. However, study results on the *curative *effect on a present depression are conflicting. A recent Cochrane meta-analysis [13], updating an earlier systematic review in 2001 by Lawlor and Hopker [14], concluded that the statistically weak findings didn't support the efficacy of exercise in the treatment of depression. Methodological weaknesses of the trials were identified, including the lack of treatment concealment, intention to treat analysis and a clinical interview to confirm the diagnosis of depression.

Rethorst et al. [15] argue that the earlier mentioned meta-analysis of Lawlor and Hopker suffered from incomplete research data and a lack of moderating variable analysis. The meta-analysis by Rethorst et al., with 58 RCTs included, calculated an overall effect size of -0.80 for the effects of exercise on depression. The recommendation of both Lawlor et al. and Rethorst et al. is that further, conclusive research is still needed into the use of exercise for depression. Exercise as an adjunct to recognised treatments, such as psychotherapy and/or medication, should also be studied. Furthermore, follow-up research is needed that examines the sustainability of the effects after exercise therapy has ended.

Very recently, Krogh et al. [16] performed a systematic review and meta-analysis in which only 13 trials were selected because of the stringent selection criteria used (i.e. recording clinical depression according to any diagnostic system, adequate allocation concealment, blinded outcome and intention to treat analysis). Besides an inverse association between duration of intervention and the magnitude of the association of exercise with depression, the effect size was 0.40 for the pooled effect sizes of 13 studies and 0.19 for the selected three high quality design studies. The authors concluded that large high quality design studies are required. The recommendations from the three meta-analysis cited above are integrated in the design of the EFFORT-D study.

Besides reduction of depressive symptoms, exercise can be useful for other treatment indications, such as cardiovascular condition, metabolic syndrome, pain modulation and the immune system. Depression is associated with *cardiovascular disease*. It is an established risk factor for mortality after acute myocardial infarction. A recent meta-analysis of cohort studies also shows the aetiological and prognostic effects of depression on coronary heart disease (CHD) [17]: the pooled relative risk of depression on future coronary heart disease was 1.80. Another meta-analysis shows an overall relative risk of dying in depressed subjects of 1.80 compared to non-depressed subjects, with no major differences between men and women [18]. The increased relative risk on mortality was also found in subclinical forms of depression.

These findings suggest that exercise may also be prescribed to improve the cardiovascular condition and prevent future heart disease. Depressed patients should also be more closely monitored regarding cardiovascular parameters.

The body of evidence showing an association between depression and *obesity and metabolic deregulation *is growing. Recent meta-analysis of cross-sectional studies in the general population found a significant positive association between depression and obesity, affecting women more than men. A reciprocal risk relation between obesity and depression [19,20], and an U-shaped, non-linear trend in the association between BMI and depression was found [21]. The Dutch NESDA study [22] shows an association between severity of depression and unfavourable cholesterol high-hdl/low-ldl concentration [23]. This tendency for a higher risk of depression on metabolic syndrome, in which various cardiovascular risk factors appear, emphasizes the need of careful screening on this parameters [24], and exercise could positively influence these parameters.

A recent meta-analysis of studies into cytokines in depressed patients [25] showed that depression and an *activation of the immune-system *do co-exist. Both tumornecrosisfactor-α (TNF-α) and interleukine-6-concentration (IL-6) were significantly raised and C-reactive protein (CRP) was linearly associated with several conventional risk factors and inflammatory markers [26]. The relevance of CRP however still remains controversial [27]. Therefore, measurement of CRP blood levels in an intervention exercise study could contribute to a better understanding of the clinical importance in relation to heart condition.

The reciprocal relation between *pain and depression *has been reported in several studies where Major Depressive Disorder (MDD) was associated with chronic pain (> 6 months) and nearly 50% of patients had at least one chronic painful physical condition [28,29]. The duration of depressive symptoms was found to be prolonged in the presence of chronic pain. The therapeutic prognosis of co-morbid pain and depression is poor and the pain-depression association was found to be stronger in men than in women and in older adults compared to younger. Productivity was decreased when depression and pain existed in a co-morbid pattern [30].

### Quality of life and cost-effectiveness

Systematic evaluation of cost-effectiveness and quality of life, including life-events in the treatment of depression, may contribute to the development of more evidence based care models [31], for instance by disease management.

A population sample study showed that, in patients with a remitted MDD, the quality of life was lower than in the general population. A higher depressive severity was associated with a lower quality of life. Ten Doesschate et al. argue that, even in depression in remission, attention is needed for the quality of life, and above that, residual symptoms must be treated aggressively to achieve a higher quality of life [32].

### Summarized aims and hypothesis of EFFORT-D

The main objective of the study is to assess the effectiveness of exercise therapy (running therapy (RT) or Nordic walking (NW)), in addition to usual care, on depression in adult patients. We hypothesize that adding exercise therapy to usual care will result in a larger reduction in depressive symptoms (as measured with the Hamilton Rating Scale of Depression, (HRSD)) during a 6 month treatment program as well as at 6 months follow-up, compared to usual care without exercise therapy.

Secondary aims are to assess the effectiveness of exercise therapy (RT or NW) on the following outcome measures: 1) Cardiovascular and metabolic risk parameters as fitness (VO2 max, grip strength), BMI, waist circumference, body composition, blood pressure, fasting blood glucose, cholesterol HDL/LDL ratio and CRP, 2) Co-morbid symptoms of anxiety and pain 3) Quality of life, and 4) Cost-effectiveness.

To assess whether there are subgroups of patients who show a larger or less effect of exercise therapy on depression, subgroup analysis will be conducted differentiated on demographic variables (age, sex) and level and type of depression based on the HRSD, personality traits and level of fitness.

## Methods/Design

**Figure 1:**
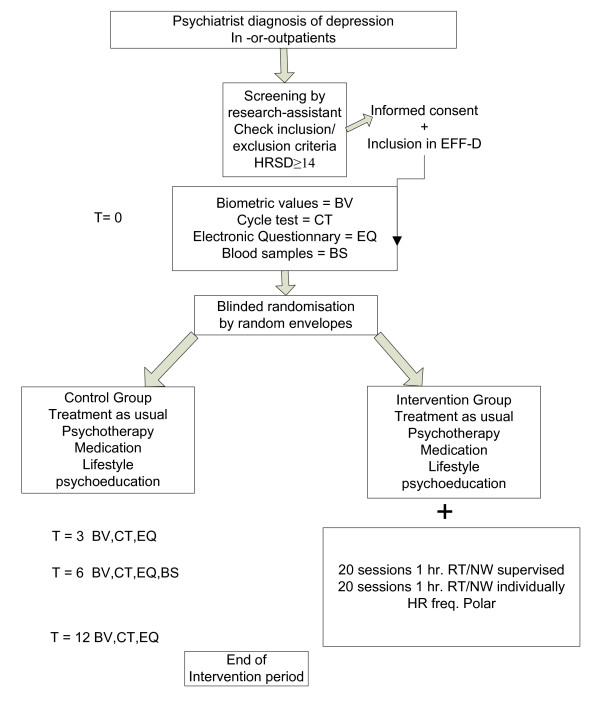
**Flow chart**.

EFFORT-D is a randomized controlled trial, designed along the Consort-statement guidelines [33], in which outpatients as well as hospitalised depressed patients will be included and randomized in two groups: a control group or an intervention group (*see flow chart in *Figure 1). The control group outpatients receive usual care (i.e. anti-depressive medication and/or cognitive and/or interpersonal therapy). The control group inpatients have their usual treatment program (consisting of pharmacotherapy and/or sociotherapy, psychotherapy, psycho-education and indicated nonverbal therapies) and are allowed to exercise at low intensity as part of their daily program.

The intervention group receives 6 months supervised exercise therapy for 1 h a week and is instructed to train unsupervised for 1 h a week during this period (combined about 40 exercise sessions during the intervention period). Both training sessions follow an individualised intervention protocol and are in addition to the usual care program.

Included and randomised patients in the intervention group are invited to take part in Running Therapy (RT). Patients will only be referred to Nordic Walking (NW) in case of clear medical contra-indications against RT, such as muscular-skeleton problems, or if patients have a strong dislike for running which obstructs participation and compliance. The study will run for 27 months, with an 18 months inclusion period during which patients are recruited and randomized. There are four measurements for each patient: at baseline (T0), halfway the 6 month intervention period (T3), at the end of the intervention period (T6), and at follow up, 12 months after baseline (T12). *In *Table 1* the timetable is shown*.

**Table 1:** Events and time table

					Intervention period			Follow-up period
**Procedure**	**Source**	**Person**	**No. of items**	**Duration (min.)**	**T0**	**T3**	**T6**	**T12**

Written informed consent	letter	patient			X			

Demographics^1^	patient file	research assistant			X			

**Depression**								

Depression history^2^	patient file	research assistant	2		X			

HRSD^3^	interview	blind rater	17	20	X	X	X	X

IDS-SR^4^	questionnaire	patient	30	10	X	X	X	X

Bearableness (VAS)	questionnaire	patient	1		X	X	X	X

**Metabolic syndrome**								

Length	physical test	research assistant		1	X			

Weight	physical test	research assistant		1	X	X	X	X

Waist circumference	physical test	research assistant		1	X	X	X	X

Blood pressure	physical test	research assistant		1	X	X	X	X

Smoking/alchohol intake	questionnaire	patient		1	X	X	X	X

Laboratory assesment^6^	physical test	laboratory		2	X	X	X	X

**Quality of Life**								

WHO-DAS^7^	questionnaire	patient	36		X	X	X	X

**Pain**								

GCPS^8^	questionnaire	patient	7		X	X	X	X

Bearableness (VAS)	questionnaire	patient	1		X	X	X	X

**Anxiety**								

BAI^9^	questionnaire	patient	21		X	X	X	X

**Cost effectiveness**								

TIC-P^10^	quest./pnt file	pnt/research assistant		26	X		X	X

Euroqol	questionnaire	patient		5	X		X	X

Subjective health (VAS)	questionnaire	patient		1	X		X	X

**Fitness**								

Submaximal cycle test	physical test	research assistant		10	X	X	X	X

Grip strength	physical test	research assistant		2	X	X	X	X

Exercise intensity (HR)^11^	physical test	exercise instructor			during training sessions			

**Personality**								

NEO PI-R^12^	questionnaire	patient	60		X			

**Physical activity**								

SQUASH^13^	questionnaire	patient	12		X	X	X	X

**Additional Measures**								

LEQ^14^	questionnaire	patient	12		X	X	X	X

Compliance	registration	exercise instructor			during training sessions			

POMS^15^	questionnaire	patient	32		during training sessions			

Evaluation Intervention^16^	questionnaire	patient					X	

1: Date of birth, sex, education, ethnicity, income, professional status, living situation, marital status

2: Number and duration of former episodes, duration current episode

3: Hamilton Rating Scale of Depression (or HAMD)

4: Inventory of Depressive Symptomatology - Self Report

6: fasting glucose, triglycerides, total cholesterol, HDL- cholesterol, cholesterol/HDL-ratio, creatinine, MDRD

7: World Health Organisation- Disability Assessment Schedule

8: Graded Chronic Pain Scale

9: Beck Anxiety Inventory

10: Trimbos/iMTA questionnaire for Costs associated with Psychiatric Illness

11: Polar RS 800 CX Run

12: Revised NEO Personality Inventory

13: Short QUestionnaire to ASses Health enhancing physical activity

14: Life Events Questionnaire

15: Profile of Mood State

16: Only for patients who participated in the intervention

### Study population

The study population consists of adult patients diagnosed by a clinician with a depression or bipolar disorder with depressive mood, who are or will be treated in GGZ Centraal Mental Hospitals or Symfora-Meander Hospital. Patients aged between 18-65, with a DSM-IV diagnoses of unipolar, bipolar depression or seasonal depression not responding to light therapy (10 sessions of 1 h), a baseline Hamilton Rating Scale of Depression (HRSD) score ≥14 and (will be) treated for depression, are included.

Criteria for exclusion are: a depression as part of a psychotic disorder, schizophrenia, schizoaffective disorder or obsessive compulsive disorder, anxiety disorder as primary diagnosis, patients in long stay facilities (including day-care) or with complex pathology and treatment resistant depression (inpatients, treated by protocol more than 6 months with no remission); patients with significant cardiovascular disease or other medical conditions as contra-indication for exercise therapy, walking and/or running such as joint and hip pathology; alcohol/drugs dependence as a primary diagnosis, pregnancy, high suicide risk with treatment on a closed ward, or already being physically active on a regular basis (2-3 times a week on a high-intensity).

### Sample size

Following earlier RCTs [34] it is expected that patients in the usual care group (controls) will respond with a mean reduction in HRSD of six points. Adding exercise to usual care is expected to result in a decline of at least eight points in HRSD score (thus two extra points). To detect this difference, with an α (two-tailed) of 5% and a power (1-β) of 80%, using two equal groups and a standard deviation of 5 points, 100 patients are needed in each group. Taking 30% drop-out into account, 140 patients have to be included in each group.

### Procedures and study instruments

Names of eligible patients with their registration number of the electronic patient file (EPD) are provided by the diagnosing psychiatrists to the research assistant, who will make a first check on inclusion and exclusion criteria, informs the participants and asks them to join the study. Written informed consent will be obtained according to prevailing legal requirements before the start of the study. Eligible patients, HDRS ≥ 14 as primary outcome measure, perform the Åstrand submaximal cycling test, physical measures and fill out the questionnaire, after which the participants are randomized. All outcome parameters measured during baseline will be repeated three, six and 12 months after baseline, except for the blood samples (only at T0 and T6). Height is measured according to protocol (Seca 214, Hamburg, Germany) and a bio-impedance scale is used to measure weight and body composition (Omron HBF-510, Omron Healthcare Europe BV, The Netherlands). Waist circumference is measured twice with a tape measure (Seca 201, Hamburg, Germany) at the midpoint between the lower border of the ribs and the upper border of the pelvis. Systolic and diastolic blood pressure are registered twice at rest, using an electronic blood pressure meter (Omron M6 comfort, Omron Healthcare Europe BV, The Netherlands) with an adequate cuff size. Grip-strength is tested according to protocol using a hydraulic hand dynamometer (Jamar J00105, Sammons Preston Rolyan, Bolingbrook, USA). The submaximal Åstrand test [35] will be performed on a stationary bicycle ergometer (Examiner, Lode BV, The Netherlands) and the mean heart rate of the last 2 min of the test will be used to estimate the VO_2_max. Heart rate during this test is registered by a heart rate monitor (Polar RS 800, Electro Oy, Finland).

### Questionnaires

At each measurement moment the participant will be asked to fill out a digital questionnaire containing the following instruments:

#### Demographics and personal life events

Socio-demographic data are collected using standard questions on age, sex, marital status, ethnicity and household composition. Socio-economic variables include highest education and income. Personal history is evaluated by the Life Events Questionnaire (LEQ), a 12-item inventory-type questionnaire in which subjects mark the exposure to negative life events such as unemployment, separation from a partner and death of a close family member which have occurred in the past year [36].

#### Mental and physical health and its consequences

The Hamilton Rating Scale of Depression (HRSD) measures depression with a 17-item list performed by trained interviewers [37]., using the Dutch translation of the version of Bech et al. [38], in which the items of depressive symptoms are extensively operationalised and this version is often used in international research [39].

The Inventory of Depressive Symptomatology - Self-Reported (IDS-SR) measures the severity of depression with a 30-item self-report list [40]. It has good responsiveness to change and is more sensitive for atypical depressive criteria than the HRSD. History of depression is evaluated by a single question into the number and duration of depressive episodes for which treatment was necessary.

Bearableness of depression is measured with a visual analogue scale (VAS) ranging from 0 (very unbearable) to 100 (very well bearable). Anxiety is measured by the Beck Anxiety Inventory (BAI), a 21-item multiple-choice self report inventory that measures the severity of generalized anxiety and panic symptoms in adults and adolescents [41].

Pain complaints are evaluated with the Graded Chronic Pain Scale (GCPS) [42], a 7-item scale measuring aspects of pain, physical ability and social interference, resulting in a 5-class hierarchical scale ranging from 0 (no pain problem) to IV (high disability/severely limiting). Next to the GCPS, bearableness of pain is evaluated with a VAS-scale ranging from 0 (very unbearable) to 100 (very well bearable).

Other secondary outcomes are disability during the last 30-days associated with both physical and mental problems and is measured by a shortened version of the World Health Organisation - Disability Assessment Schedule II (WHO-DAS-II) [43], resulting in a disability score ranging from 0-100 with a higher score reflecting greater disability. Quality of life data are collected using the EQ5D [44], a standardized instrument for describing and valuing health related quality of life.

Subjective health is evaluated by a visual analogue scale ranging from 0 (the worst imaginable health condition) to 100 (the best imaginable health condition).

Health care use and work productivity are evaluated by the Trimbos/iMTA Questionnaire for Costs associated with Psychiatric Illness (TIC-P), a 29-item list which focuses on establishing costs related to loss of productivity at work and health care utilization [45].

##### Variables expected to modify the effect of the intervention

Personality, as a possible effect modifier, is measured at baseline by the NEO-PI-R, a 60-item validated questionnaire measuring the five domains of personality including neuroticism, extraversion, agreeableness, conscientiousness and openness to experience [46].

##### Confounders: Lifestyle behaviours

Self-reported level of physical activity is assessed by means of the validated Short QUestionnaire to ASsess Health enhancing physical activity (SQUASH), a 12-item questionnaire which evaluates the frequency and duration of physical activities in the domains of work, domestic and leisure time [47]. Tobacco and alcohol intake is measured with a standard single question on the frequency of use per day.

#### Additional measures in the intervention group

The Profile of Mood States (POMS) registers participants' mood after a running session for in total three times during the intervention period (at the beginning, halfway and at the end). The Dutch shortened version of the POMS [48] consists of 32 items divided over seven subscales including tension, depression, anger, fatigue, vigour, positive and negative affect.

At regular intervals participants score their exertion on a Borg-scale ranging from 6 (very, very light) to 20 (very, very severe) [49]. At the end of the intervention period, or when participants dropped out of the RT or NW therapy, a short questionnaire is administered evaluating their satisfaction and experience with the intervention.

### Randomisation, blinding and treatment allocation

Randomisation takes place at every location separately. This way, every location will have an equal distribution of participants between the intervention and control group. The SPSS random generator (SPSS version 14.0) [50] will be used to allocate patients. Ten closed envelopes with allocation numbers are presented to the participants. They choose an envelope, after which the research assistant tells the patients in which arm of the study they are included. Evaluators of the main outcome measure (HRSD) are blinded for group allocation and are trained regularly for inter-rater reliability. All other measures will be evaluated by a research assistant, who is not blinded for group allocation.

### Exercise intervention

The exercise sessions will take place twice a week (40 sessions in total): once a week a supervised group session is offered and once a week the patient does an individual training, with clear instructions beforehand and an evaluation at the beginning of the next supervised session. Each supervised session, in which the trainers are working according to a standardized protocol, lasts one hour, of which 30 min are spent running (RT)/Nordic walking (NW). The remaining time is spent on warming-up and cooling-down. Each patient follows an individualised intervention protocol with a gradually increasing training intensity. The goal is to achieve a 30 min period of continuous running in the last sessions (two times a week 30 min continuous aerobic exercise at least 60% of the maximal heart rate). The NW program follows a comparable progressive schedule with increased time spent Nordic walking with high intensity.

Intensity in RT as in NW is monitored by the instructor during every supervised session by counting the heart rate and three times during the intervention period by electronic registration (Polar RS 400, Electro Oy, Finland). The control group receives usual care for depression in accordance with the revised Dutch guideline [51] and are advised to exercise regularly. Hospitalised and day-care patients in the control group are supposed not to participate in organised high intensity aerobic exercise during the intervention period. Only low-intensity activity psycho-motor therapy is allowed.

### Compliance and withdrawal

In order to improve compliance during the intervention period, a protocol will be followed concerning missed exercise therapy sessions by participants. This protocol includes: 1) active approach by the exercise instructor in case of no show, and 2) encouragement of other participants to contact each other in case of no show. Participants can withdraw at any time for any reason without any consequences. Also, the investigator can decide to withdraw a participant from the study for urgent medical reasons. Participants who withdraw from the intervention will be asked the reason(s) for drop-out but will be retained in the study for the intention to treat analysis.

### Statistical analyses

Comparability of the intervention and control groups will be examined for the baseline measurements. If necessary, analyses will be adjusted for baseline differences. The primary analysis of the data set will be according to the 'intention to treat' principle. A secondary 'per protocol' analysis will be done taking into account the level of compliance and the amount of exercise during the intervention period. Usual daily physical activity, tobacco smoking and alcohol intake will be treated as confounders.

Differences in remission rates (and other categorical outcomes) between the experimental groups are examined by contingency table Chi-square statistics. Differences in mean scores on continuous outcomes (e.g. HRSD) between the intervention groups are examined by analysis-of-variance.

### Ethical principles and safety

The study has been designed and will be carried out in accordance with the principles of the Helsinki Declaration (Edinburgh, Scotland Amendment, October 2000). The study protocol has been approved by the Medical ethical committee for mental health (Metigg Kamer Noord).

## Discussion

The aim of this study is to investigate the effectiveness of aerobic exercise therapy (RT or NW) on depression in adult patients in addition to usual care. Also, the effect of physical exercise on frequently existing co-morbid diseases or risk factors for such disorders as metabolic syndrome is an objective of this study.

This is the first well conducted add-on randomised controlled high-quality trial into the effect of aerobic exercise on depression with a correct randomisation procedure, blinded outcome assessment, intention to treat analysis, study into cost-effectiveness, quality of life and long-term follow-up as was advised in earlier publications. EFFORT-D can therefore contribute to stronger evidence for this type of intervention, which can result in more specified recommendations for clinical practice. Another strength of this study is the fact that also severely clinically depressed patients, who are mostly excluded in other studies, will be included. A relatively greater effect of exercise in this subgroup of severely depressed patients is possible. A challenge of the study lies in the motivational techniques needed to exercise with depressed patients, which is proven to be difficult [52] and it will take a serious effort not to exceed the calculated 30% drop-out patients in the intervention group. Because this study is supported by a strong hypothesis and minimal negative side effects are expected, one-tailed statistical analysis is also possible if the large number of included participants can't be reached within the planned time. Furthermore, the diversity of outcome measures makes it possible to distinguish more explicitly those depressed patients that could benefit most from exercise.

## Competing interests

The authors declare that they have no competing interests.

## Authors' contributions

FRK is principle investigator, psychiatrist and project leader of the project in GGZ Centraal and Symfora-Meander Hospitals and drafted the manuscript, IH is project leader for the study at Body@Work, designed the study and has been involved in drafting the manuscript, ET was involved in the study design, organizing the data and commented the manuscript, AJB was involved in the study design and revised the manuscript critically and gave approval for publication, MHR was involved in the study design, revised the manuscript critically and gave final approval of the version to be published.

## Pre-publication history

The pre-publication history for this paper can be accessed here:

http://www.biomedcentral.com/1471-2458/12/50/prepub
